# Integrated analysis of tumor microenvironment features to establish a diagnostic model for papillary thyroid cancer using bulk and single-cell RNA sequencing technology

**DOI:** 10.1007/s00432-023-05420-8

**Published:** 2023-09-21

**Authors:** Yizeng Wang, Wenbin Song, Yingxi Li, Zhaoyi Liu, Ke Zhao, Lanning Jia, Xiaoning Wang, Ruoyu Jiang, Yao Tian, Xianghui He

**Affiliations:** 1 Department of General Surgery, Tianjin Medical University General Hospital, 154 Anshan Road, Heping District, Tianjin, 300052 People’s Republic of China; 2 Key Laboratory of Immune Microenvironment and Disease (Ministry of Education), Tianjin Medical University, Tianjin, 300070 People’s Republic of China

**Keywords:** Papillary thyroid cancer, Biomarker, Diagnostic model, Single-cell RNA sequencing, Tumor microenvironment

## Abstract

**Background:**

Characterizing tumor microenvironment using single-cell RNA sequencing has been a promising strategy for cancer diagnosis and treatment. However, a few studies have focused on diagnosing papillary thyroid cancer (PTC) through this technology. Therefore, our study explored tumor microenvironment (TME) features and identified potential biomarkers to establish a diagnostic model for papillary thyroid cancer.

**Methods:**

The cell types were identified using the markers from the CellMarker database and published research. The CellChat package was conducted to analyze the cell–cell interaction. The SCEVAN package was used to identify malignant thyroid cells. The SCP package was used to perform multiple single-cell downstream analyses, such as GSEA analysis, enrichment analysis, pseudotime trajectory analysis, and differential expression analysis. The diagnostic model of PTC was estimated using the calibration curves, receiver operating characteristic curves, and decision curve analysis. RT-qPCR was performed to validate the expression of candidate genes in human papillary thyroid samples.

**Results:**

Eight cell types were identified in the scRNA-seq dataset by published cell markers. Extensive cell–cell interactions like FN1/ITGB1 existed in PTC tissues. We identified 26 critical genes related to PTC progression. Further, eight subgroups of PTC tumor cells were identified and exhibited high heterogeneity. The MDK/LRP1, MDK/ALK, GAS6/MERTK, and GAS6/AXL were identified as potential ligand-receptor pairs involved in the interactions between fibroblasts/endothelial cells and tumor cells. Eventually, the diagnostic model constructed by TRPC5, TENM1, NELL2, DMD, SLC35F3, and AUTS2 showed a good efficiency for distinguishing the PTC and normal tissues.

**Conclusions:**

Our study comprehensively characterized the tumor microenvironment in papillary thyroid cancer. Through combined analysis with bulk RNA-seq, six potential diagnostic biomarkers were identified and validated. The diagnostic model we constructed was a promising tool for PTC diagnosis. Our findings provide new insights into the heterogeneity of thyroid cancer and the theoretical basis for diagnosing thyroid cancer.

**Supplementary Information:**

The online version contains supplementary material available at 10.1007/s00432-023-05420-8.

## Introduction

Thyroid cancer (THCA) is one of the most common endocrine tumors globally (Xia et al. 2022; Siegel et al. 2023). Papillary thyroid cancer (PTC)—a pathological type of THCA—accounts for approximately 90% of THCA patients (Fagin and Wells 2016; Chen et al. 2023). Ultrasound is currently the standard technique for evaluating the characteristics of thyroid nodules. Existing risk stratification systems classify the risk of malignant tumors according to the ultrasonic findings of thyroid nodules (Kobaly et al. 2022). Although 10–15% of nodules eventually prove malignant, 5% of patients have distant metastases at diagnosis (Alexander and Cibas 2022). About 15% of patients treated with surgery relapse during postoperative follow-up, leading to a poor prognosis (Ito et al. 2018; Alexander and Cibas 2022; Fallahi et al. 2022). Even after postoperative radioactive iodine therapy, some patients develop refractory PTC. With a better understanding of the molecular progression of THCA, several drugs have been developed for inhibiting oncogenic kinases or signaling kinases (RET/PTC, *BRAF* (V600E)), like those related to vascular endothelial growth factor receptor platelet-derived growth factor receptor and platelet-derived growth factor receptor (Fallahi et al. 2022). *BRAF* (V600E) mutations have been confirmed to be associated with poor prognosis of PTC patients (Costa et al. 2008; Song et al. 2021). Unfortunately, PTC patients without *BRAF* mutation could not benefit from these newly developed drugs. The effect of *BRAF* inhibitors in non-*BRAF* mutated cancers has reportedly been controversial (Agianian and Gavathiotis 2018). Therefore, future research must delve into the molecular heterogeneity of PTC to find novel diagnostic biomarkers and provide patients with individualized treatment.

With the development of sequencing technology, researchers are able to observe the cellular level changes in tumor tissue though single-cell RNA sequencing (scRNA-seq) technology, which brings new strategies for tumor diagnosis and treatment (Hwang et al. 2018). The tumor microenvironment, a daedal ecosystem comprise stromal, epithelial, and immune cells (Xiao and Yu 2021). Different immune infiltrating state in the tumor microenvironment is related to distinct prognostic outcomes in tumor patients. For instance, the presence of tumor-associated fibroblast is correlated with worse outcomes in gastric, bladder, and breast cancers (Bartoschek et al. 2018; Chen et al. 2020; Li et al. 2022). New markers and therapeutic targets have been developed by further exploring the tumor microenvironment. Sui et al. (2023) revealed the role of the *CCL18*/*PITPNM3* ligand-receptor pair in the interaction between tumor cells and macrophages by single-cell data of esophageal squamous cell carcinoma, and the receptor could be used as a potential therapeutic target for esophageal cancer. Ma et al. (2020) found the heterogeneity of prostate tumor cells, and *HPN* could be used as an early-stage diagnostic marker for prostate cancer by analyzing single-cell data of prostate cancer. Therefore, a deep understanding of the tumor microenvironment of PTC helps to elucidate the underlying mechanisms of tumor progression and occurrence, as well as to search for new biomarkers and potential therapeutic targets.

The present study extensively characterized the tumor microenvironment of PTC and established a diagnostic model-based six genes. Our findings revealed extensive *FN1*/*ITGB1* communication between the T/NK and other cells in PTC compared to normal thyroid tissue. Furthermore, we found dynamic changes in the evolution of thyroid cells into tumor cells and a high degree of heterogeneity among tumor cells. *MDK/ALK/ALP* and *GAS/MERTK/AXL* interactions between tumor cells and endothelial cells/fibroblasts may be potential therapeutic targets for PTC. Finally, we established a diagnostic model with good diagnostic efficacy for PTC by combining bulk RNA-seq. Our findings provided novel insights into the diagnosis of PTC.

## Materials and methods

### Data collection and data preprocessing

A total of 665 samples were included in the present study: 7 scRNA-seq samples (1 normal thyroid sample and 3 pairs of bilateral PTC) from the Gene Expression Omnibus (GEO) cohort (GSE191288, https://www.ncbi.nlm.nih.gov/geo/query/acc.cgi?acc=GSE191288); 564 RNA-seq data (505 PTC and 59 normal samples) from The Cancer Genome Atlas (TCGA) cohort; 94 RNA-seq data (49 PTC and 45 normal samples) from GEO cohort. (GSE33630, https://www.ncbi.nlm.nih.gov/geo/query/acc.cgi?acc=GSE33630). The gene expression profiles of the TCGA dataset were downloaded using the TCGAbiolinks package (v2.26.0), then converted to TPM format and standardized with log2.

The “Seurat” R package (v4.3.0) was used to conduct quality control procedures and downstream bioinformatics analyses for scRNA-seq datasets. We used the following criteria to obtain high-quality cells and filter out low-quality cells: the proportion of mitochondrial genes counts ≤ 15%; the proportion of erythrocyte genes counts ≤ 3%; UMIs ≥ 500; genes detected per cell ≥ 200. The DoubletFinder (McGinnis et al. 2019) package was used to remove the doublets in each sample. After removing the doublets, 28,587 cells were included in further research. Subsequently, we normalized the scRNA-seq expression through the glmGamPoi (Ahlmann-Eltze and Huber 2021) package. The top 3000 highly variable genes were identified using the SCTransform method and then used to calculate principal components. The TSNE and UMAP methods in Seurat were performed for cell clustering. The Clustree package was utilized to find the optimal cluster resolution. The markers used for cell identity were obtained from published research and the CellMarker database (Zhang et al. 2019; Pu et al. 2021).

### Cell–cell interaction analysis

The CellChat (Jin et al. 2021) package was used to evaluate the difference in cell–cell interaction between the normal and THCA samples. The two data sets were normalized separately, the high-variable genes were identified, and PCA analysis was performed in each of the two gene sets using the high-variable genes. Anchors were identified with the findinintegrationanchors() function, and the IntegrateData() function was used to combine two data sets. Additionally, the cell–cell interaction analysis among 16 cell types was also performed according to standard procedures. *P* value < 0.05 was considered statistically significant.

### CNV analysis

SCEVAN—an R package—can infer non-malignant and malignant cells in the tumor microenvironment by calculating the raw count matrix of scRNA data. The thyrocyte cell expression matrix of six tumor samples (N1L, N1R, N2L, N2R, N3L, and N3R) were extracted and performed copy number variation (CNV) analysis to determine malignant thyrocyte cells with the SCEVAN package.

### Single-cell downstream analysis

The SCP package (https://github.com/zhanghao-njmu/SCP) provides a comprehensive set of tools for single-cell data downstream analysis. The present study used SCP to perform multiple single-cell downstream analyses, such as pseudo-time trajectory analysis, enrichment analysis, GSEA analysis, and differential expression analysis. In detail, the trajectory analysis was performed by RunSlingshot() function in SCP to infer the evolution of thyrocytes into tumor cells. The tumor cells were extracted and regrouped into eight groups. Enrichment analysis between tumor groups was performed using the RunGSEA() function in SCP, and an adjusted *P* value < 0.05 was considered statistically significant. Furthermore, RunDEtest() function in SCP was used to identify the over-expression genes in eight distinct groups of tumor cells. Genes with log2FC > 1 and *P* value < 0.05 were considered over-expressed genes. Gene Ontology (GO) and Kyoto Encyclopedia of Genes and Genomes (KEGG) enrichment analyses were performed to annotate the biological processes over-expressed genes are involved in.

### Clinical relevance

Differential expression analysis between thyrocytes and tumor cells was performed using the RunDEtest() function in SCP. We obtained 22 markers with log2FC > 1 and *P* value < 0.05. A total of 1186 genes up-regulated in PTC tissue were calculated by edgeR package. In detail, differential methods were set as ANOVA, and genes with log2FC > 1 and *P* value < 0.05 were considered up-regulated genes. The intersection of the aforementioned genes led to the identification of eleven genes. Boruta and LASSO method were utilized to identify the most important genes.

Logistic regression was used to construct a diagnostic model with important genes to better predict the thyrocyte tissue type. Receiver operating characteristic (ROC) curves were utilized to assess the discriminative performance. The calibration curves were applied to estimate the predictive accuracy of the model using the bootstrap method with 1000 re-samplings. To better explore model fitting, Hosmer–Lemeshow (HL) tests were performed, and *P* value > 0.05 was recognized as a good model fitting. The clinical applicability of the diagnostic model was estimated by the decision curve analysis (DCA). The TCGA dataset was used as training cohort, and GEO dataset was used as testing cohort.

### RT-qPCR

Total RNA was extracted using TRIzol Reagent (Invitrogen, Carlsbad, CA, USA) from frozen THCA tissue and corresponding control tissue resected surgically according to the manufacturer’s recommendations. RNA quality and concentration were measured using the NanoDrop 2000 spectrophotometer (Thermo Scientific, USA). Real-time polymerase chain reaction (PCR) reactions were performed using SYBR Green PCR Master Mix (TransGen Biotech, Beijing, China) on the 7500 Real-Time PCR System (Applied Biosystems, Waltham, MA, USA). The specific sequences of primers are listed in Additional file 1: Table S1.

### Statistical analyses

The RT-qPCR results were statistically analyzed using Prism 8 (Graph pad Software, CA) and are presented as mean ± standard deviation (SD) for at least three individual experiments. The statistical significance of differences was determined with the unpaired, two-tailed student *t* test, and *P* < 0.05 was considered statistically significant. Other statistical analyses in this study were performed using R studio software 4.2.2

## Results

### Identification of cell types

**Fig. 1:**
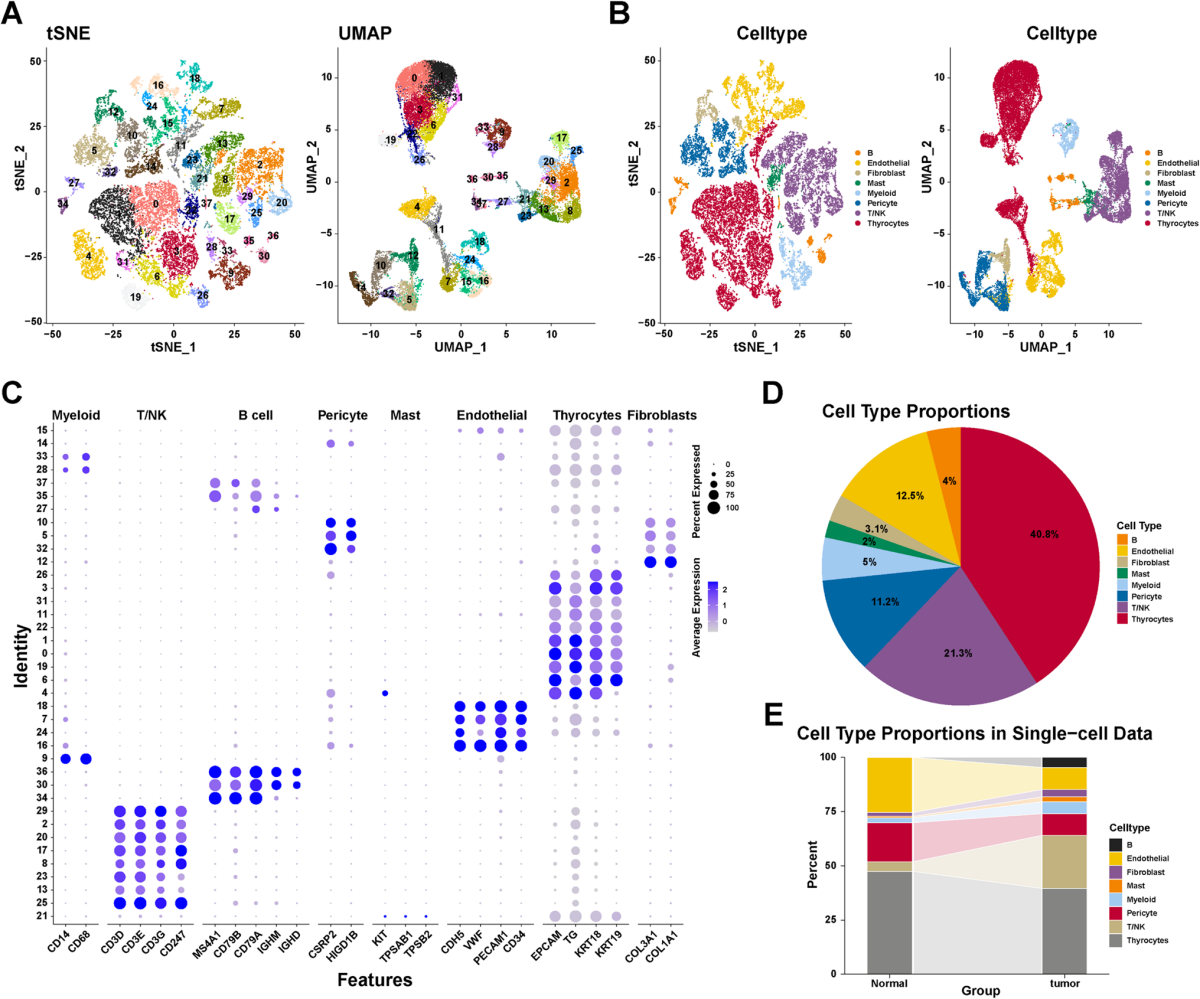
Distinct cell types in PTC were identified through single-cell sequencing. The cell clusters (**A**) and cell types (**B**) in PTC tissue demonstrated using the uniform manifold approximation and projection (UMAP) and t-distributed stochastic neighbor embedding (TSNE) plots according to their featured gene expression profiles. **C** Dot plot displaying the expression level of marker genes for annotating the cell types.** D** The cell type portions of the whole scRNA-seq dataset **E** The cell type portions of normal and tumor samples in scRNA-seq dataset

A total of 28,587 cells were obtained after quality control procedures through Seurat. All cells in the scRNA-seq dataset were clustered into 38 clusters using PCA with a resolution of 1. As shown in Fig. 1A, two-dimensionality reduction methods—TSNE and UMAP—revealed that different cell clusters were separated. We further annotated the cell types with cell markers from the Cell Marker database and published research. All cells were further annotated into eight cell types: T/NK cells, B cells, endothelial cells, myeloid cells, fibroblasts, mast cells, pericytes, and thyrocytes (Fig. 1B). The used cell markers are shown in Fig. 1C. For example, *CD3D*, *CD3E*, *CD3G,* and *CD247* are markers of T/NK cells, and nine-cell clusters (2, 8, 13, 17, 20, 21, 23, 25, and 29) are identified as T/NK cells. These results indicated that the used markers could clearly distinguish different cell types. Furthermore, Fig. 1D shows the proportion of different cell types: thyrocytes (40.8%), T/NK cells (21.3%), pericytes (11.2%), myeloid cells (5%), endothelial cells (12.5%), fibroblasts (3.1%), B cells (4%), and mast cells (2%). Figure 1E depicts the proportion of cell types in normal and tumor samples. The proportion of non-epithelial cells in tumor samples is higher than that in normal samples, indicating a more complex microenvironment of tumor samples. These results revealed the characteristics of the data set and the differences in cell proportions between the samples. Taken together, we identified eight main cell types for further exploration.

### Communication network difference between the normal and thyroid cancer tissue

**Fig. 2:**
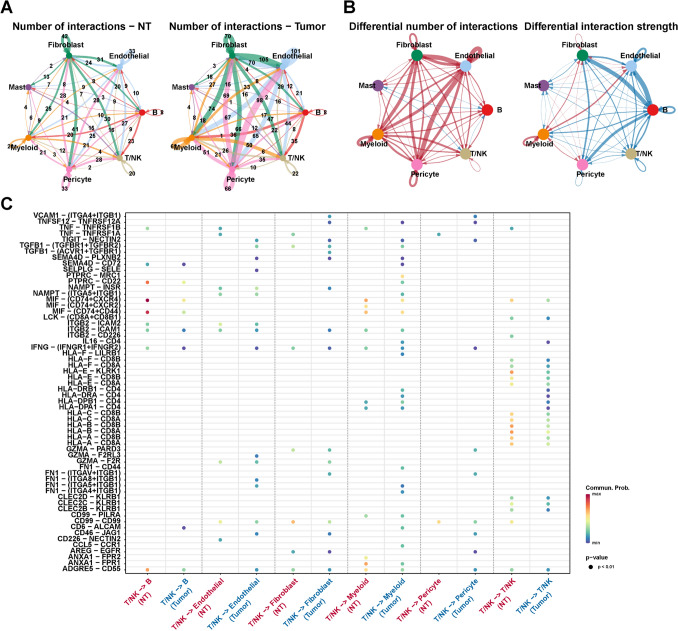
Cell–cell interaction differences in tumor and normal tissues. **A** Interaction net count plot of non-tumor (NT) and tumor tissues. The thicker the line represented, the more the number of interactions between the two cell types. Number represents the number of interactions. **B** Interaction net count and strength plot of tumor tissues compared with those of NT tissue. The thicker the line represented, the more the number of interactions, and the stronger the interaction weights/strength between the two cell types. Red represents tumor tissues. Blue represents NT tissue.** C** Dot plot exhibited the differences in ligand-receptor pairs between the T/NK and other cells in NT and tumor tissues

Cell communication analysis can infer cell–cell interactions to further explore the changes in the tumor microenvironment. To analyze the changes in cell–cell interactions between normal and PTC tissues, we performed the cell communication analysis among seven cell types, excluding thyrocytes, using CellChat. Figure 2A depicts the number of interactions among seven cell types between normal (NT) and tumor samples, with cell–cell interactions in tumor tissues more than that in normal tissue. Subsequently, we compared the number and intensity of interactions between tumors and normal tissue (Fig. 2B). Compared to the normal sample, the number and intensity of interactions among endothelial cells, myeloid cells, and fibroblasts were higher in tumor tissues (N1L, N1R, N2L, N2R, N3L, N3R), suggesting the crucial role of three cell types in the formation and development of the PTC tumor microenvironment.

Considering the significance of T/NK cells in the TME, we further analyzed the interactions between T/NK and other cells, including themselves. For ligand-receptor pairs such as *FN1*-*ITGB1*, the probability of T/NK cells interacting with endothelial cells, fibroblasts, and myelocytes in PTC tissue was significantly higher than that in non-tumor tissue (Fig. 2C). This result suggested that *FNI*/*ITGB1* could play a critical role in the tumor microenvironment of PTC. These findings reveal the potential role of extensive cellular communication in promoting tumor microenvironment formation in PTC tissue.

### The cell trajectory of PTC epithelial cells

**Fig. 3:**
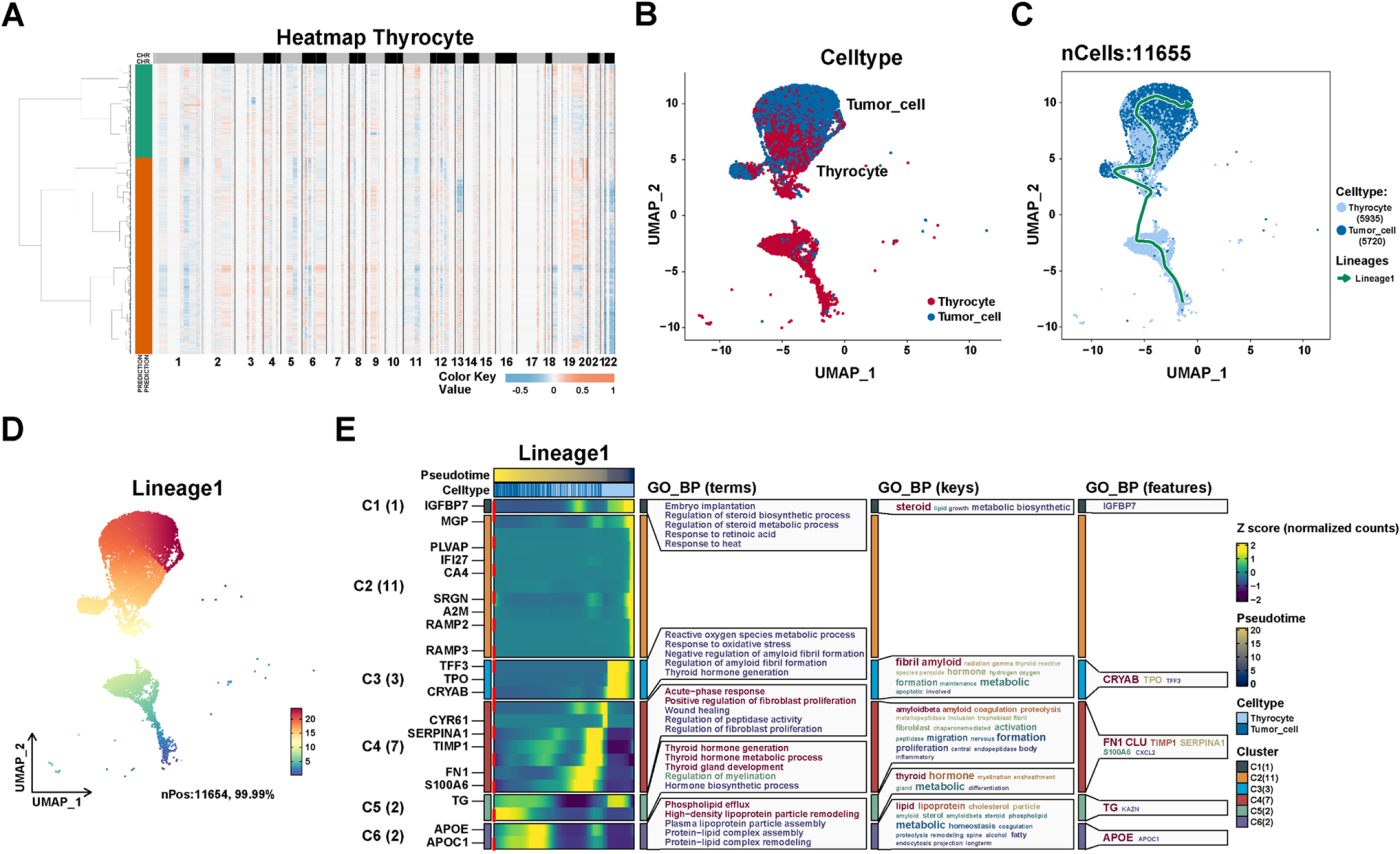
Reconstructing the pseudotime trajectory of tumor cells using thyrocytes and tumor cells and identifying genes varied during the trajectory. **A** heatmap of CNV levels in thyrocytes. Green represents normal thyrocytes, and yellow represents malignant thyroid (tumor) cells in legend. Color key from deep blue to yellow indicates relative CNV levels from low to high. **B** Cell type assignment following UMAP-based visualization of expression differences for 11,655 single thyrocytes (normal and malignant thyrocytes) from 7 samples in the scRNA-seq dataset. **C** Cell trajectory of normal thyrocytes and tumor cells was generated using a slingshot in SCP. Lineages represented cell trajectory directions.** D** Pseudo-time is colored in a gradient from blue to red. The start of pseudo-time is indicated by blue, whereas the end of pseudo-time by red. **E** The differential expressed genes (DEGs) with expression levels that changed the most over the pseudo-time trajectory were divided into six clusters based on their expression trend, and the representative processes of each cluster are shown. Color key from deep blue to yellow indicates relative expression levels of the DEGs from low to high. The numbers in parentheses after the cluster represent the trees of the gene

As not all epithelial cells in tumor tissues are malignant, SCEVAN was used to infer the benign and malignant nature of epithelial cells in tumor tissues. SCEVAN was used to analyze copy number variation (CNV) on 9577 thyrocytes from tumor tissues (N1L, N1R, N2L, N2R, N3L, and N3R). As shown in Fig. 3A, 5720 tumor cells were identified with abnormal levels of CNV compared to thyrocytes. Subsequently, we extracted 11,655 thyrocytes and tumor cells in 7 samples (1 NT and 6 tumor samples) for further analysis (Fig. 3B).

To investigate the dynamic development of epithelial cells in PTC microenvironment, we performed the cell trajectory analysis with the slingshot method in the SCP package to infer the trajectory from thyrocytes to tumor cells. As shown in Figs. 3C, D, thyrocytes were shown at the beginning of the trajectory, whereas tumor cells were located in the end of trajectory. To further explore the critical genes and biological progress in PTC progression, we analyzed the dynamic changes of genes in the trajectory “lineage 1”. As shown in Fig. 3E, the most important genes involved in PTC progression were identified: *IGFBP7*, *MGP*, *PLVAP*, *IFI27*, *CA4*, *SRGN*, *A2M*, *RAMP3*, *RAMP4*, *TFF3*, *TPO*, *CRYAB*, *CYR61*, *SERPINA1*, *TIMP1*, *FN1*, *S100A6*, *TG*, *APOE*, and *APOC1*. Additionally, the biological progress, including dysregulation of steroid metabolic process, dysregulation of thyroid hormone metabolic process, and positive regulation of fibroblast proliferation, were potentially involved in PTC progression. These findings reveal the dynamics of the biological processes that occur on the trajectory from thyrocytes to tumor cells. Furthermore, these genes closely related to tumor cell development may serve as potential biomarkers for PTC progression.

### The heterogeneity between thyroid tumor cells

**Fig. 4:**
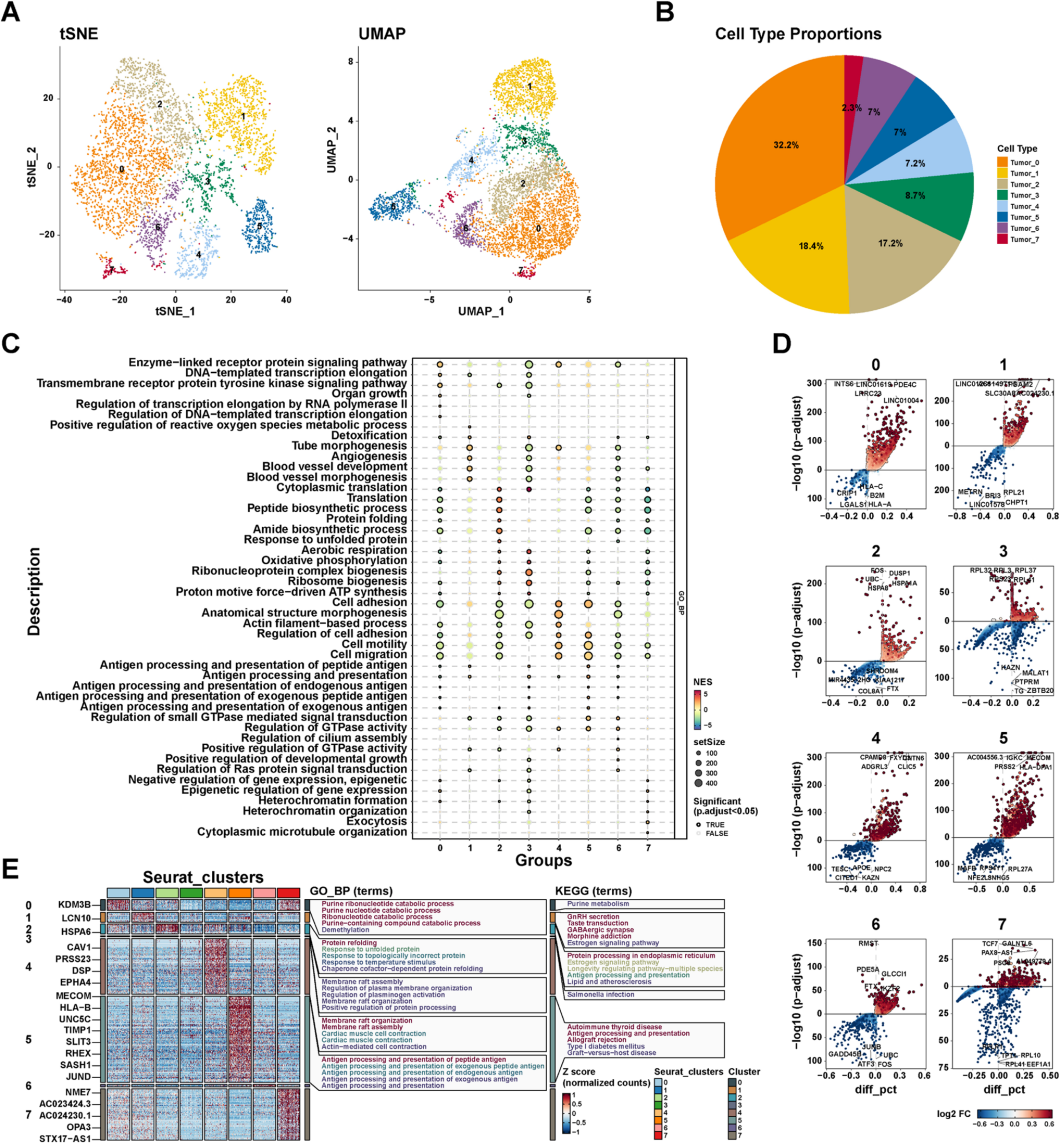
Subgroups in tumor cells were sub-clustered using PCA. **A** eight subgroups generated from tumor cells are demonstrated using tSNE and UMAP. **B** Statistics of cell percentage of each subgroup in tumor cells. **C** Dot plot showing the representative biological processes enriched in each subgroup. **D** The volcano plots show differential expressed genes of each subgroup. The horizontal axis represents log2-fold change of genes. The vertical axis represents − log10 (*P* adjust value). **E** The heatmap shows up-regulated genes of each subgroup, and the representative processes and KEGG pathways of each cluster are shown. Color from blue to red indicates relative expression levels of the genes from low to high

To further explore the heterogeneity among thyroid tumor cells, we re-clustered 5720 tumor cells using the same procedures and the SCTransform method. As shown in Fig. 4A, B, tumor cells were divided into eight groups with PCA. We performed gene set enrichment analysis (GSEA) based on each group compared to other groups. Compared with other groups, subgroup 0 of tumor cells was related to enzyme-linked receptor protein signaling pathway, organ growth, and DNA-templated transcription elongation (Fig. 4C). Subgroup 1 of tumor cells was mainly involved in tube morphogenesis, angiogenesis, blood vessel development, and morphogenesis, suggesting that this subgroup may be involved in angiogenesis in the tumor microenvironment. Subgroups 2 and 3 were associated with cytoplasmic translation, oxidative phosphorylation, and ribosome biogenesis compared with other groups (Fig. 4C). Similarly, Subgroups 4 and 5 were correlated with cell adhesion, cell motility, and cell migration (Fig. 4C), indicating the potential role of Subgroups 4 and 5 in tumor metastasis. In addition, Subgroups 6 and 7 were also involved in different biological processes (Fig. 4C). These findings indicate the characteristics of tumor subgroups at the level of the biological processes involved and the role of each subgroup in the TME.

We further analyzed the differential genes of each subpopulation compared to other subpopulations (Fig. 4D). The genes with logFC > 1 and *P* value < 0.05 were considered markers of each group (Table S2). As shown in Fig. 4E, the up-regulated genes of subgroup 0 were related to purine metabolism. The up-regulated genes of Subgroups 1 and 2 were involved in estrogen signaling pathways. Additionally, antigen processing and presentation were up-regulated in subgroups 2 and 5. Additionality, subgroup 5 was also associated with autoimmune thyroid disease. These findings reveal the heterogeneity of thyroid tumor cells, including intercellular metabolism, immunity, and tumor-related signaling pathways.

### The crosstalk of tumor cells with other cells in the TME

**Fig. 5:**
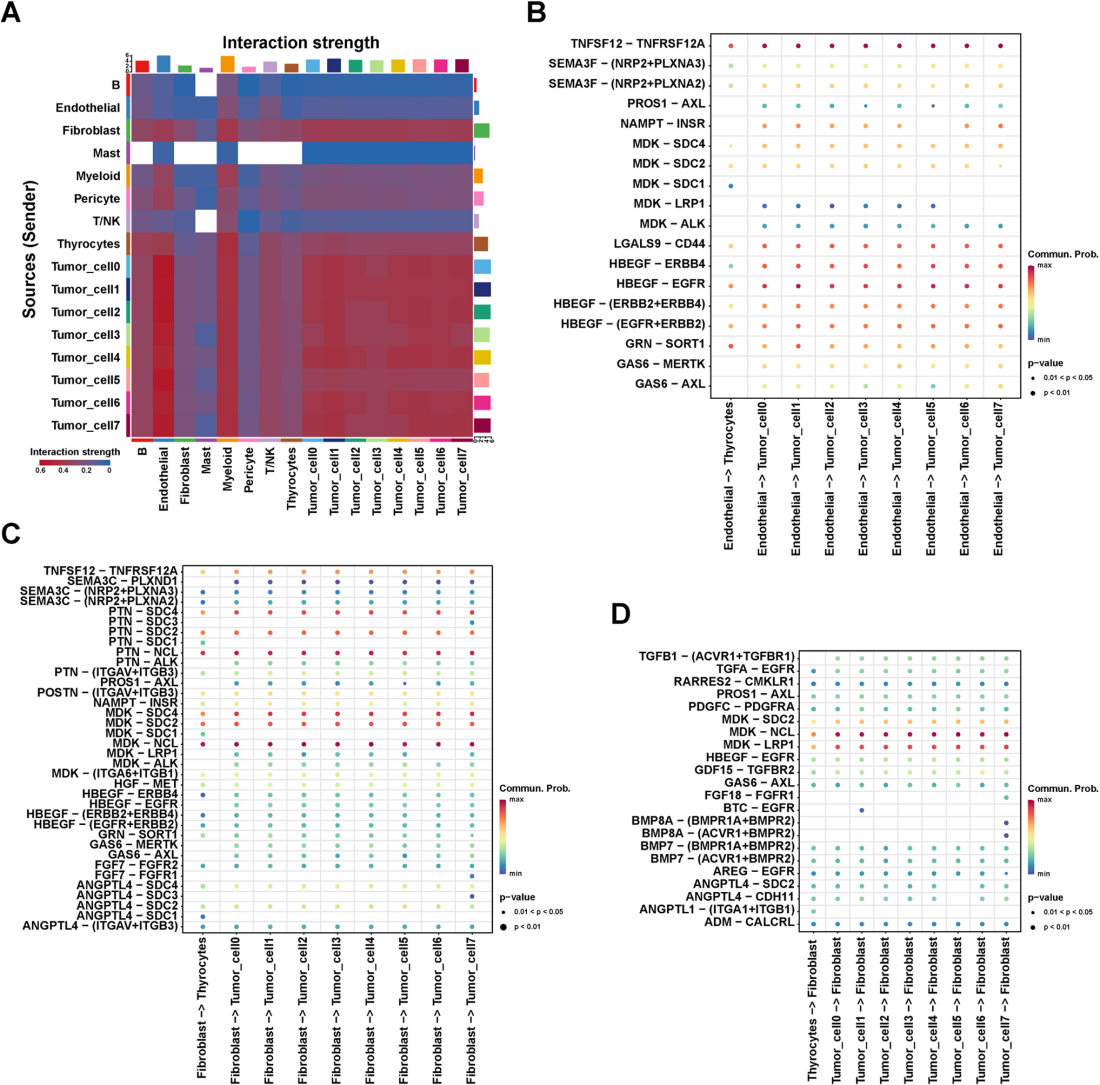
The crosstalk between tumor cells and other cells. **A** Heatmap shows the interaction strength among all cells in PTC. Color from blue to red indicates the interaction strength of two cell types from low to high. Blank space represents no significance. Dot plots show potential ligand-receptor pairs for endothelial interaction with tumor cells (**B**), fibroblast interaction with tumor cells (**C**), and tumor cell interaction with fibroblast (**D**). Color from blue to red indicates relative interaction probability from low to high. Blank space represents no significance

To further explore the role of tumor cells in the TME, we analyzed all cell–cell interactions using CellChat. As shown in Fig. 5A, the interaction strength between tumor cell subgroups 0–7 and endothelial cells was highest, implying that the endothelial cells may be crucial in promoting tumor cell growth. Additionally, the interaction strength of fibroblasts on tumor cells was significantly enhanced compared with thyrocytes (Fig. 5A). Therefore, we further analyzed the ligand–receptor pair interaction between endothelial cells, thyrocytes, and tumor cells. Ligand–receptor pairs, such as *PROS1-AXL*, *NAMPT-INSR*, *MDK-LRP1*, *MDK-ALK*, *GAS6-MERTK*, and *GAS6-AXL*, between endothelial and tumor cells exhibited a higher communication possibility than that between endothelial cells and thyrocytes (Fig. 5B). These results revealed the potential interactions between tumor cells and endothelial cells in PTC.

Notably, *MDK/LRP1*, *MDK/ALK*, *GAS6/MERTK*, and *GAS6/AXL* between fibroblasts and tumor cells exhibited a higher communication possibility than that between fibroblasts and thyrocytes (Fig. 5C). In view of our previous findings in cell trajectory analysis that genes that positively regulate fibroblast growth were up-regulated in tumor cells, we analyzed the interactions of thyroid cells and tumor cells on fibroblasts. The probability of interaction between tumor cells and fibroblast receptor-ligand pairs was higher than that of thyroid cells, especially in *TGFB1/ACVR1/TGFBR1*, *FGF18/FGFR1*, *BTC/EGFR*, *BMP8A/BMPR1A/BMPR2*, and *BMP8A/BMPR1A/BMPR2* (Fig. 5D). Consistent with the results of cell trajectory analysis, these results suggested that tumor cells could also have a regulatory effect on fibroblast growth via the aforementioned ligand-receptor pairs. Overall, these results revealed the potential role of endothelial cells and fibroblasts in regulating tumor cell growth in the tumor microenvironment.

### Identification of critical diagnostic genes for PTC

**Fig. 6:**
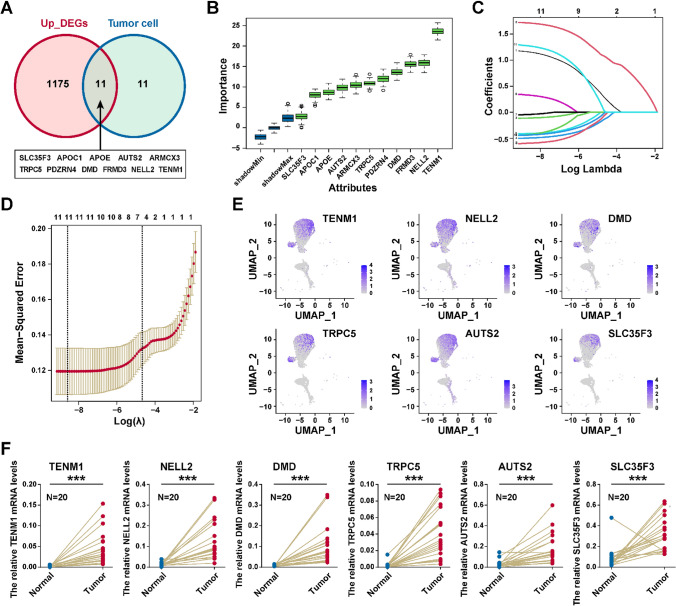
Identification of candidate diagnostic genes.** A** Venn diagram showing the intersection of up-related genes in tumor tissue from the TGCA-THCA (PTC) dataset and marker genes of tumor cells from the scRNA-seq dataset. **B** Importance score of candidate genes calculating by Boruta algorithm. **C** LASSO regression analysis: coefficient values at varying levels of penalty. Each curve represents a gene. **D** Ten-fold cross-validation was used to calculate the best lambda, contributing to the minimum mean cross-validated error. **E** Expression levels of six candidate diagnostic genes plotted onto the UMAP in the scRNA-seq dataset. Color key from gray to blue indicates relative expression levels from low to high. **F** The mRNA relative expression of seven candidate diagnostic genes in normal and cancerous thyroid tissues. *P < 0.05, **P < 0.01, ***P < 0.001

To identify the critical diagnostic genes for PTC, we performed the differential expression gene analysis between thyrocytes and tumor cells in the scRNA-seq dataset, and 22 markers of tumor cells were identified (Table S3). Next, we performed the differential expression gene analysis between TCGA-THCA (PTC samples) and normal samples, and 1186 up-regulated genes in PTC were obtained (Table S4). After intersection of these two gene sets, we firstly identified eleven genes (*TRPC5*, *TENM1*, *PDZRN4*, *NELL2*, *FRMD3*, *APOE*, *ARMCX3*, *DMD*, *APOC1*, *SLC35F3*, *AUTS2*) highly expressed at both the tumor cell and tissue levels (Fig. 6A). Boruta package was used to identify importance of genes, and all eleven genes were confirmed as critical genes (Fig. 6B). We next performed LASSO method to identify six critical genes (*TRPC5*, *TENM1*, *NELL2*, *DMD*, *SLC35F3*, and *AUTS2*; Fig. 6C, D). As shown in Fig. 6E, these candidate diagnostic genes were obviously overexpressed in the tumor cells. Finally, the RT-qPCR analysis demonstrated that *TRPC5*, *TENM1*, *NELL2*, *DMD*, *SLC35F3*, and *AUTS2* were highly expressed in PTC tissue than in normal tissue (Fig. 6F). In summary, *TRPC5*, *TENM1*, *NELL2*, *DMD*, *SLC35F3*, and *AUTS2* could be potential biomarkers for PTC diagnosis.

### Construction of a diagnostic model for PTC

**Fig. 7:**
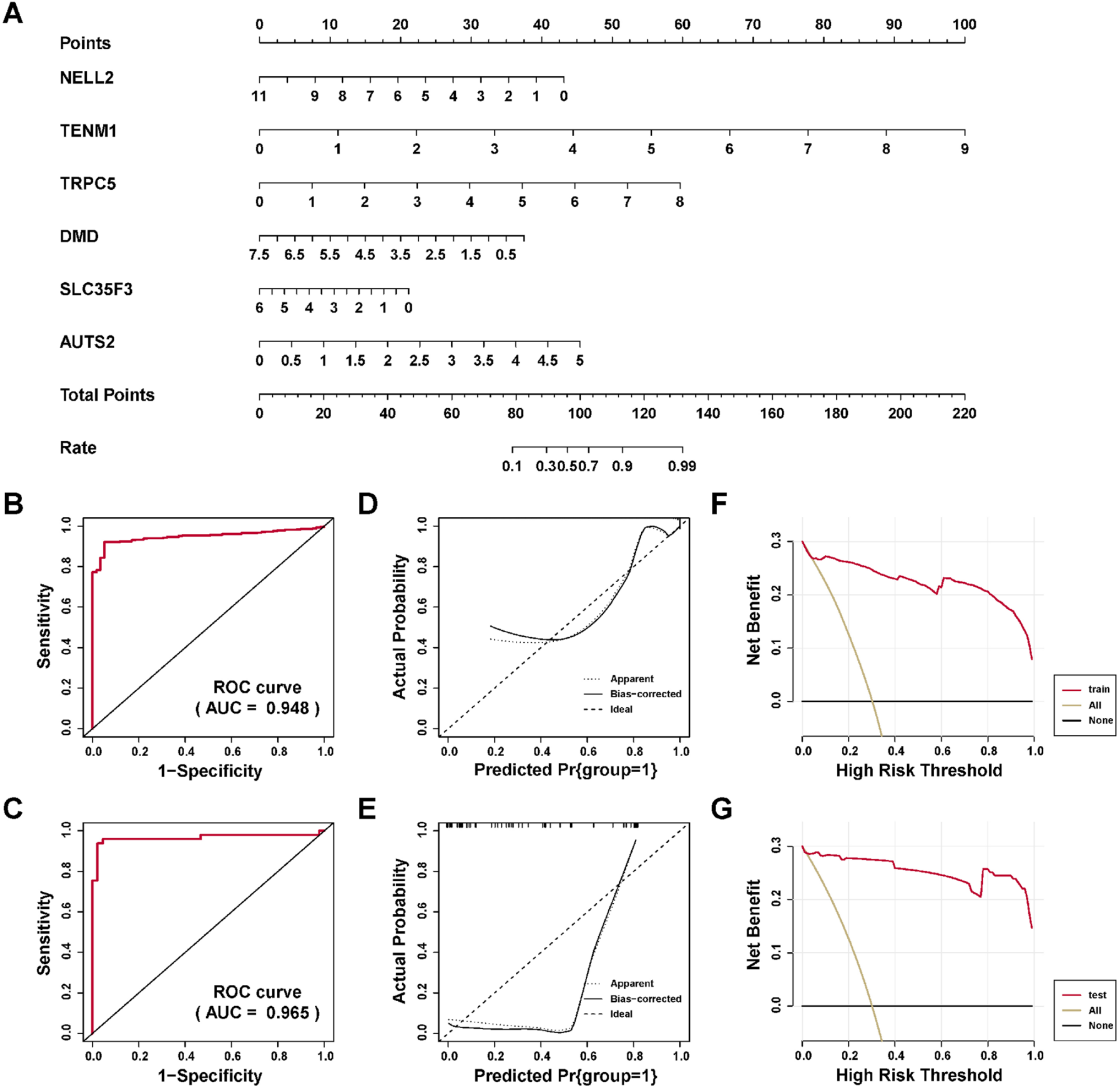
Construction and validation of a diagnostic model for PTC.** A** Nomogram to estimate the risk of PTC tissues. The area under ROC curve was utilized to estimate the discrimination of the model in training set (**B**) and test set (**C**). Calibration curves of the diagnostic model in the **D** training (HL test, *P* = 0.94) and **E** validation cohorts (HL test, *P* = 0.14). DCA of the diagnostic model in the **F** train and **G** test cohorts

To construct a diagnostic model for diagnosis for PTC, logistic regression was performed to construct a nomogram to predict the risk of PTC based on six critical genes (Fig. 7A). ROC R package was utilized to assess the discriminatory ability of the diagnostic model. The AUCs were 0.948 (Fig. 7B) and 0.965 (Fig. 7C) in the train and test sets, respectively, suggesting the effective role of the model to distinguish the PTC and normal tissues. In addition, the calibration curves in the train and test set suggested good predictive accuracy (Fig. 7D, E), with a *P* value for HL tests were 0.94 and 0.14 in train and test sets. Finally, the DCA were performed to analyze the clinical benefit of the model we constructed. As shown in Fig. 7F, G, the model we established could confirm the good benefits of clinical intervention. In a word, our model with good discriminatory ability could be served as a potential tool for PTC diagnosis.

## Discussion

A large number of transcriptomic and genomic studies have been performed to identify diagnostic and prognostic biomarkers for THCA. Several effective diagnostic and prognostic biomarkers are prepared to apply in clinical practice for THCA, including somatic mutations and other molecular changes (Nikiforov and Nikiforova 2011). However, PTC, as a main type of THCA, is a highly heterogeneous cancer. Previous studies have explored RNA expression in tumor tissue based on transcriptomics, which could not accurately reflect differences in gene expression between different cells within the tissue. Single-cell sequencing has emerged to identify individual-cell-level changes in gene expression. Therefore, with the progress of sequencing technology, we can better characterize the tumor microenvironment of PTC and find more specific tumor-related biomarkers for precise diagnosis and individual treatment.

Here, we comprehensively analyzed the tumor microenvironment of PTC. Our results revealed the potential aberrant interactions between the T/NK and other cells (endothelial cells, fibroblasts, and myeloid cells) through *FN1/ITGB1*. Fibronectin is confirmed to be a critical role of wound healing, cellular differentiation and growth, migration, and adhesion (Rick et al. 2019). And integrin‐β (*ITGB*), a member of the integrin superfamily, is essential for cell proliferation, adhesion, and differentiation (Miranti and Brugge 2002). Gu et al. (2023) found that overexpression of *ITGB1* in gastric cancer was related to a poor prognosis and immune suppression. Also, increased *ITGB1* expression was confirmed to be associated with poor prognosis and increased fibroblast infiltration in pancreatic ductal adenocarcinoma (Benesch et al. 2022). Consistently with *ITGB1*, high *FN1* expression was also related to M2 macrophage infiltration and poor prognosis in THCA (Geng et al. 2021). These findings suggested the immune suppression role of FN1/ITGB1 in tumor microenvironment. Taken together, T/NK cells in PTC may form the immunosuppressive tumor microenvironment via *FN1/ITGB1* interaction with other cells. Therefore, *FN1/ITGB1* may be a potential therapeutic target to target the tumor microenvironment in PTC.

Through the CNV analysis, we identified thyroid tumor cells and analyzed the potential trajectories of thyroid cell differentiation into tumor cells. Several genes related to tumor cell differentiation were identified: *CYR61*, *SERPINA1*, *TIMP1*, *FN1*, *S100A6*, *APOE*, and *APOC1*. Studies have confirmed the prognostic values of *CYR61* (Ren et al. 2021), SERPINA1 (Wu et al. 2021), and *FN1* in THCA. By further subdividing the tumor cells, eight distinct tumor subgroups were identified. Tumor subgroups were involved in different biological processes, indicating a high degree of heterogeneity among tumor subgroups. The cell–cell interaction analysis revealed a strong interaction possibility between tumor cells and fibroblast/endothelial cells. Cancer-associated fibroblasts (CAFs) play essential roles in tumor development. They secrete extracellular matrix proteins, inflammatory ligands, and growth factors which promote cancer cell proliferation, immune exclusion, and therapy resistance (Biffi and Tuveson 2021). Fibroblasts and endothelial cells are the primary sources of CAFs. Endothelial cells are involved in intravasation, which allows invasive cancer cells to translocate into the blood vessel lumen (Sobierajska et al. 2020). These studies have revealed the critical role of endothelial cells and fibroblasts in TME. Further, our results identified potential ligand-receptor pairs (*MDK/LRP1*, *MDK/ALK*, *GAS6/MERTK*, and *GAS6/AXL*) for cell communication between these two types of cells and tumor cells. The *MDK* and *GAS* signaling pathway has been confirmed to regulate several biological processes in cells, including proliferation, survival and migration in tumor microenvironment by binding to their receptors (Wu et al. 2018; Filippou et al. 2020). Therefore, endothelial cells and fibroblasts may regulate the development of tumor cells and promote tumor microenvironment though these pairs.

To identify effective diagnostic markers, we intersected the up-regulated genes in tumor cells and up-regulated genes in PTC tissues. *TRPC5*, *TENM1*, *NELL2*, *DMD*, *SLC35F3*, and *AUTS2* were identified as diagnostic biomarkers for THCA. To validate our findings, we performed RT-qPCR on 20 cases of paired PTC and para-cancer tissue. These gene expression levels confirmed the accuracy of our analysis. Although further study and clinical experiment are needed, the potential of the six genes to be successful diagnostic biomarkers for PTC has been consolidated in published studies. For example, Kechin et al. (2022) found that *AUTS2* might be as one of important genes which can classify PTC in relation to the presence of driver NTRK-chimeric TRK genes. In addition, AUTS2 was also reported to be positively associated with other cancer progression via TGF-beta pathway activation, HEDGEHOG and WNT signaling pathway (Han et al. 2015). These pathways play an essential role in tumor metabolism and immunity (Hanna and Shevde 2016; Zhao et al. 2020; Zou and Park 2023). Cheng et al. (2017) reported that *TEMN1* was highly expressed in cancerous thyroid tissues, and *TENM1* expression in PTC was related to an advanced stage, *BRAF* V600E mutation, extra-thyroidal invasion and the classical subtype. These findings demonstrate the diagnostic efficacy and potential of candidate genes in differentiating benign and malignant thyroid tissue. In addition, the diagnostic model we constructed shows good differentiation and accuracy in both train and external test set, which indicates that the model is stable and practical. However, our study has some limitations. First, the scRNA-seq data in our study was limited. More single-cell data must be collected to reveal the prevalence of microenvironmental features in PTC. Additionally, in vitro and in vivo experiments are needed to further verify the mechanism of the receptor–ligand we screened for in PTC. Finally, we just analyzed and validated the mRNA levels of six candidate biomarkers; their application must be confirmed through further experiments and clinical studies.

In conclusion, we characterized the tumor microenvironment of thyroid papillary carcinoma and identified essential receptor and ligand pairs. Notably, we comprehensively explored the heterogeneity of thyroid tumor cells. Further, we established a diagnostic model based on six candidate diagnostic markers for PTC. Our findings provide new insights into the heterogeneity of PTC and the theoretical basis for diagnosing PTC.

## Supplementary Information

Below is the link to the electronic supplementary material.

Supplementary file1 (CSV 1 KB)

Supplementary file2 (CSV 20 KB)

Supplementary file3 (CSV 2 KB)

Supplementary file4 (CSV 57 KB)

## Data Availability

Datasets related to this article are from public database (https://www.ncbi.nlm.nih.gov/geo/query/acc.cgi?acc=GSE191288, https://www.ncbi.nlm.nih.gov/geo/query/acc.cgi?acc=GSE33630). All data generated or analyzed during this study are included in this article and its supplementary information files.

## Abbreviations

THCA: Thyroid cancer
PTC: Papillary thyroid carcinoma
TCGA: The Cancer Genome Atlas
PCA: Principal component analysis
GEO: Gene Expression Omnibus
ScRNA: Single-cell RNA
CAFs: Cancer-associated fibroblasts
GO: Gene Ontology
KEGG: Kyoto Encyclopedia of Genes and Genomes
GSEA: Gene set enrichment analysis
NT: Normal tissue
TME: Tumor microenvironment
